# Closure of iatrogenic large mucosal and full-thickness defects of the stomach with endoscopic interrupted sutures in *in vivo* porcine models: are they durable enough?

**DOI:** 10.1186/s12876-015-0230-5

**Published:** 2015-01-22

**Authors:** Masakuni Kobayashi, Kazuki Sumiyama, Yamato Ban, Akira Dobashi, Tomohiko Richard Ohya, Daisuke Aizawa, Shinichi Hirooka, Kiyokazu Nakajima, Hisao Tajiri

**Affiliations:** 1 Division of Gastroenterology & Hepatology, Department of Internal Medicine, The Jikei University School of Medicine, 3-25-8, Nishi-shimbashi, Minato-ku, Tokyo, 105-8461 Japan; 2 Department of Endoscopy, The Jikei University School of Medicine, 3-25-8, Nishi-shimbashi, Minato-ku, Tokyo, 105-8461 Japan; 3 Department of Pathology, The Jikei University School of Medicine, 3-25-8, Nishi-shimbashi, Minato-ku, Tokyo, 105-8461 Japan; 4 Division of Next Generation Endoscopic Intervention, Osaka University, 2-2, Yamadaoka, Suita, Osaka 565-0871 Japan

**Keywords:** Endoscopic full thickness resection (EFTR), Endoscopic mucosal dissection (ESD), Endoscopic suturing device

## Abstract

**Background:**

In this study, we evaluated the technical feasibility of mucosal approximation of large ulcers via an endoscopic suturing system after endoscopic submucosal dissection (ESD), assessed the durability of these sutures, and compared this technique with serosal apposition of full-thickness gastric wall defects using the same device.

**Methods:**

Post-ESD ulcers were closed with mucosal apposition in 7 pigs, and endoscopic full-thickness resection (EFTR) defects were closed with serosal apposition in 3 pigs. Pigs recovered for 1 week; they were then euthanized and necropsies were performed.

**Results:**

Primary defect closure was achieved in 85.7% of the post-ESD closures and in 100% of the post-EFTR closures (*p* = 0.67). All pigs survived for 1 week. At necropsy, sutures had loosened in the post-ESD animals, although only minor deformity of the ulcer edges was observed in all repaired post-ESD ulcers. Meanwhile, all of the post-EFTR defect closures were sustained for 1 week.

**Conclusions:**

Primary closure of post-therapeutic defects can be accomplished using the device. Inverted serosal apposition provides a more durable and reliable repair than everted mucosal apposition.

## Background

Since the development of polypectomy, an array of endoscopic resection (ER) techniques have been developed with the goal of enabling larger specimens to be obtained from the mucosal layer. The endoscopic mucosal resection technique is now commonly practiced worldwide as a minimally invasive treatment for early neoplastic gastrointestinal lesions and an alternative to surgery [1]. The endoscopic submucosal dissection (ESD) technique has also been developed in an effort to eventually eliminate size as a technical limitation in endoscopically resecting lesions. Although the ability to obtain larger tissue samples made possible with ESD has succeeded in increasing the R0 resection rate and reducing local recurrence [2,3], it has inevitably led to a higher risk of post-procedure adverse events, such as bleeding and perforation [4]. While urgent surgical repair used to be the standard response to any type of iatrogenic perforation, the vast majority of perforations occurring during ER can now be managed immediately and endoscopically with the application of endoclips [5]. It is even possible to repair a gastric perforation larger than a few centimeters by sealing it with omentum and securing it with multiple endoclips [6]. However, severe delayed complications still often require surgical repair or intensive care and can even be fatal [7]. In an effort to reduce complications, prophylactic clip closure of post-ER ulcerations is now routinely practiced with the intent of preventing delayed adverse events after colonic polypectomy [8–10]. A few reports have demonstrated the technical feasibility of the mucosal closure of large post-ESD ulcers. However, as of yet, there is still debate regarding whether mucosal apposition of post-ER ulcerations affects the prevalence of delayed events [5].

**Figure 1:**
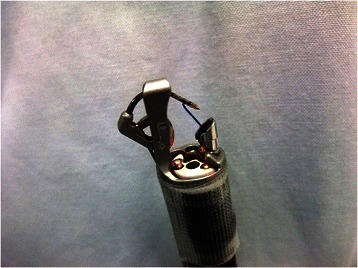
**OverStitch™ endoscopic suturing system.** The device was mounted on the tip of a two-channel gastroscope; it has a curved suturing needle.

Endoscopic full-thickness tissue apposition devices, such as endoscopic suturing systems, have been developed in an effort to achieve more robust and durable tissue approximation equivalent to that provided by surgical stitching. The OverStitch™ (Apollo Endosurgery Inc., Austin, TX) is one of these novel endoscopic suturing devices. This system provides robust tissue approximation and better control of the depth of suture placement, equal to that of surgical hand-suturing, by using a curved suturing needle (Figure 1) [11–15]. Although a series of previous publications has demonstrated that endoscopic full-thickness resection (EFTR) defects and intractable fistulas can be securely repaired with full-thickness tissue apposition devices [11–22], it is still unknown how long the sutures remain in place and whether superficial mucosal suturing is as durable as a full-thickness closure. In this 1-week survival study using a porcine model, we evaluated the technical feasibility of mucosal approximation of large post-ESD ulcers using the OverStitch™, assessed the durability of these sutures, and compared this technique to serosal apposition of full-thickness gastric wall defects using the same device.

## Methods

The protocol for the current study involving 10 pigs was approved by the Institutional Animal Care and Use Committee of The Jikei University School of Medicine. Pigs weighed 29–35 kg (mean, 33 kg). We attempted to close post-ESD ulcers and post-EFTR defects using an endoscopic suturing device. The post-ESD ulcers were closed with mucosal tissue apposition (post-ESD group) and the post-EFTR defects were closed with full-thickness tissue apposition (post-EFTR group). Seven pigs were used for post-ESD ulcer repair, and 3 were used for post-EFTR repair. All endoscopic procedures were performed by two expert endoscopists.

### Surgical preparation

The animals were fasted for 24 hours prior to the procedure. Preanesthetic medication with intramuscular midazolam (0.2 mg/kg; Dormicum; Astellas Pharma Inc., Tokyo, Japan) and medetomidine (40 μg/kg; Domitor; Nippon Zenyaku Kogyo Co., Ltd., Fukushima, Japan) was followed by the intravenous administration of propofol (2.0 mg/kg; Diprivan; AstraZeneca PLC, Tokyo, Japan). Tracheal intubation of all animals was then performed, and general anesthesia was maintained with inhalation of 1–3% isoflurane (Forane; Abbott Japan Co., Ltd., Tokyo, Japan).

### ESD technique (post-ESD group)

A therapeutic gastroscope with two accessory channels (GIF-2 T240; Olympus Medical Systems Co., Tokyo, Japan) was inserted into the stomach via an overtube (OverTube™ Endoscopic Access System; Apollo Endosurgery Inc.). First, the stomach was carefully lavaged with water. Subsequently, focal cauterization was circumferentially applied to create a tentative lesion. Two lesions of over 30 mm in maximum diameter were made in the lower half of each animal’s stomach using a needle knife (Dual Knife; Olympus Medical Systems Co.). Following the creation of a submucosal bleb by injecting saline containing 0.004% indigo carmine dye into the submucosal layer, ESD was performed in the standard manner using two types of ESD knives (the Dual Knife and IT-2 Knife; Olympus Medical Systems Co.). In 1 of the 7 pigs, a third ESD ulcer was also created in the upper corpus and left open as a control lesion.

### EFTR technique (post-EFTR group)

**Figure 2:**
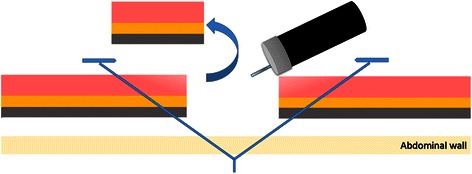
**Schematic presentation of EFTR.** T-bar style tissue anchors lifted the anterior gastric wall; a full-thickness incision was made in the tissue inside of the placed tissue anchors.

**Figure 3:**
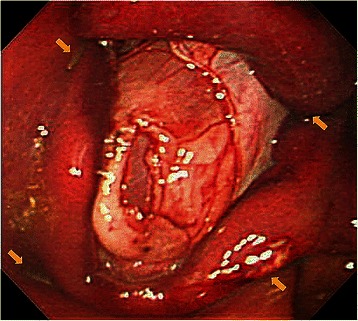
**Endoscopic image of a post-EFTR defect.** The anterior gastric wall had been tented with the tissue anchors at four points (arrows), and the defect was sealed by the abdominal wall.

EFTR was also performed with a two-channel therapeutic gastroscope via an overtube. First, digital palpation was used to identify safe sites for needle puncture (as with percutaneous endoscopic gastrostomy placement); the anterior gastric wall was then fixed to the abdominal wall at four points with T-bar style tissue anchors (Lesion lifting device; Sumitomo Bakelite Co., Ltd., Tokyo, Japan). Each tissue anchor consisted of a stainless steel rod and a wire preloaded within a 12-gauge needle and a flexible plastic outer sheath. The anterior abdominal and gastric walls were simultaneously punctured by the needle in a single stroke, and the rod was deployed into the gastric lumen. While lifting the anterior gastric wall with the anchors, an area greater than 30 mm in diameter was circumferentially incised full-thickness in the tented gastric wall inside of the four tissue anchors. Finally, the specimen was removed per os (Figures 2 and 3).

### Endoscopic suturing technique

**Figure 4:**
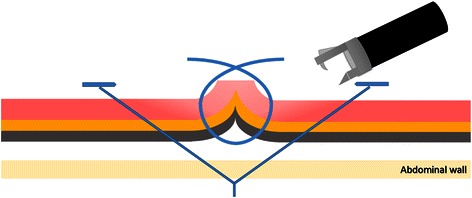
**Schematic illustrating repair of the post-EFTR defects.** The defects were closed with serosal apposition while inverting the serosal edges into the luminal side.

Closure of the defects was performed with the OverStitch™ system. Although this system is designed to be attached on the tip of the Olympus GIF 2 T-160, the Olympus 160 series scope is not available in Japan. Therefore, the device was securely taped on the tip of a GIF 2 T-240. The suture used in this study was non-absorbable and made of polypropylene. Absorbable polydioxanone suture is now available for the OverStitch™ system and might be more appropriate for the applications tested in this study, but it was not available when the study was conducted. All post-ESD and EFTR defects were completely approximated with interrupted sutures that were placed every 5–10 mm immediately after tissue removal. The post-ESD ulcers were closed with mucosal tissue apposition, and the post-EFTR defects were closed with serosal apposition while inverting the serosal edges into the luminal side. For post-EFTR closures, full-thickness penetration of the suturing needle was endoscopically confirmed at every suture placement to ensure serosal apposition (Figure 4). To evacuate excessive intra-abdominal air, an 18-gauge needle (Nipro Co., Osaka, Japan) was percutaneously placed in the anterior abdominal wall.

### Survival period

All pigs were maintained for 1 week after ESD and EFTR. Proton pump inhibitors (20 mg/day; Omeprazole; Eisai Co., Ltd., Tokyo, Japan) were orally administered for 1 week and antibiotics (500 mg/day; Cefaclor; Shionogi & Co., Ltd., Tokyo, Japan) for 3 days after the procedure. Clinically significant symptoms such as loss of appetite, tarry stool, and ptyalism were all recorded. The animals were euthanized with pentobarbital (100 mg/kg; Somnopentyl; Kyoritsu Seiyaku Co., Tokyo, Japan) after endoscopic observation of the study sites. Necropsy was performed to evaluate these sites, and all study sites were sampled and histologically evaluated.

### Outcome measures

The main outcome measure of this study was the success rate for primary closure of the gastric wall defects immediately after specimen sampling. Primary closure was determined a success when the mucosal edges of the gastric wall defects were completely approximated with the sutures. In cases of successful primary closure, secondary outcome measures were as follows: the maximum diameter (mm) of the sampled specimens; the time required for the closure, which was defined as the time elapsed from initiation of the first suture placement through the end of closure; the number of sutures applied; any clinically significant adverse events; and the success rate of secondary closure, which was determined a success when the ulcer was fused with sutures in place or bridging tissues were present at the follow-up endoscopy and necropsy.

### Statistical analysis

Data are expressed as means (± standard deviation), medians, or frequency counts (proportions). The Mann–Whitney U-test or Fisher’s exact test was used, with *p* < 0.05 considered significant. Statistical analysis was conducted with Stata 12 (Stata Corp LP College Station, TX, USA).

## Results and discussion

**Figure 5:**
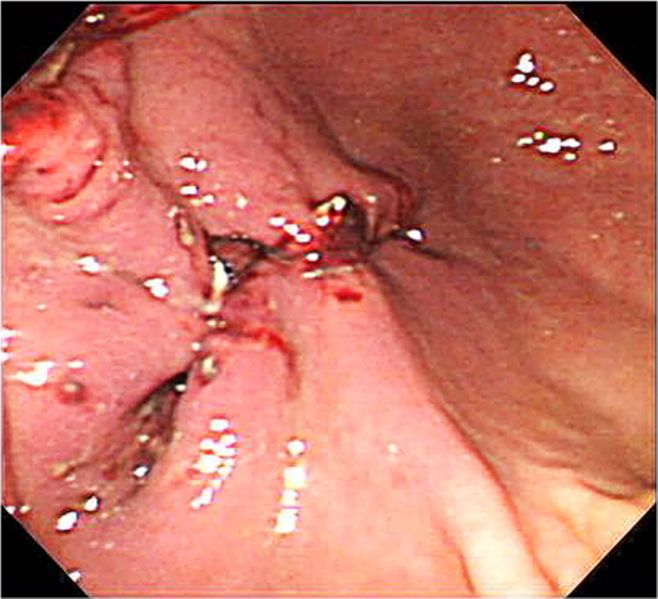
**Endoscopic findings immediately after closure in the post-ESD group.**

**Figure 6:**
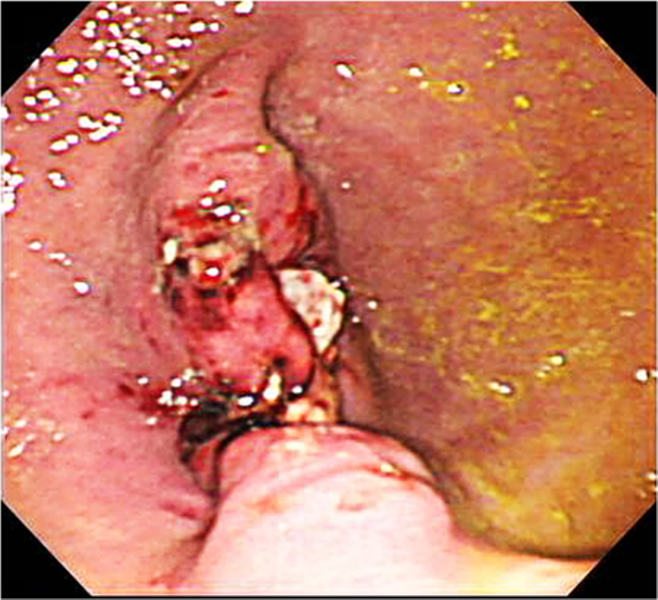
**Endoscopic findings immediately after closure in the post-EFTR group.**

**Figure 7:**
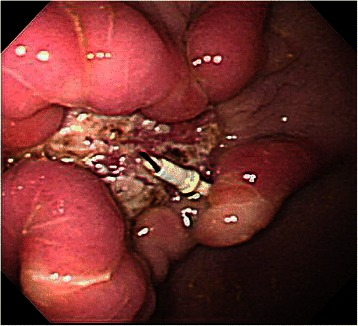
**A post-ESD ulcer at the end of the survival period (1 week after ESD).** The sutures used to repair the post-ESD ulcers had loosened and the ulcer floors were exposed in all cases.

**Figure 8:**
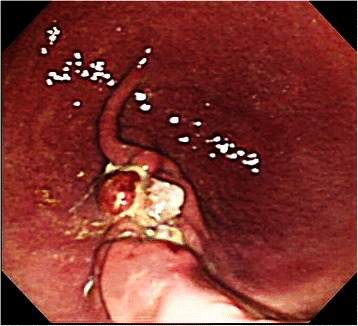
**A post-EFTR defect at the end of the survival period (1 week after EFTR).** The closures were sustained for one week in all cases.

**Figure 9:**
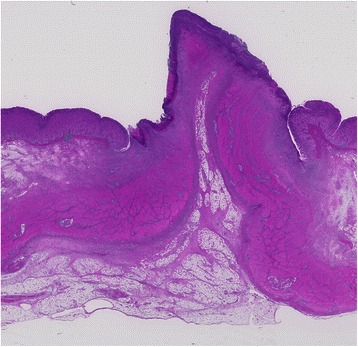
**Histological findings of a post-EFTR defect at necropsy (hematoxylin and eosin staining).** Inverted serosal apposition was maintained even after the 1-week survival period.

In this study, all pigs survived the 1-week survival period. Successful primary closure was achieved in 85.7% (12/14) of the lesions in the post-ESD group (Figure 5) and 100% (3/3) in the post-EFTR group (*p* = 0.67; Figure 6). Except for two post-ESD ulcers in the first animal to undergo the procedure, primary defect closure was achieved during all attempts. In the cases with successful primary closure (n = 12 for the post-ESD group and n = 3 for the post-EFTR group), the mean maximum diameter of sampled ESD specimens was 45.0 ± 9.3 mm (range, 35–65 mm); the mean maximum diameter of sampled EFTR specimens was 31.6 ± 2.4 mm (range, 30–35 mm; *p* = 0.015). The median procedure time was 15.5 ± 10.0 minutes (range, 7–40 minutes) in the post-ESD group and 74.0 ± 22.6 minutes (range, 35–89 minutes) in the post-EFTR group (*p* = 0.02), while the median number of sutures required to close the defects was 2.0 ± 0.5 (range, 2–3 sutures) in the post-ESD group and 4 sutures in the post-EFTR group (*p* = 0.005). In the post-ESD animal with unsuccessful primary ulcer repair, tarry stool was observed for 4 days. The successful secondary closure rate was 0% (0/12) in the post-ESD group (Figure 7) and 100% (3/3) in the post-EFTR group (*p* = 0.002; Table 1, Figure 8). In the post-ESD group, follow-up endoscopy demonstrated that the majority of the sutures barely hung on to one edge of the ulcers, but that the sutures were intact, and that the ulcers were wide open with irregular polygonal contours. The control post-ESD ulcer remained open with a sharp round contour. Meanwhile, it was histologically confirmed that the reduction in defect size established by serosal approximation after EFTR was sustained for 1 week (Figure 9). At necropsy, no surrounding organ injury due to the sutures was observed in any of the pigs. However, minor abscesses and adhesions were observed in 2 of the 3 animals (66%) in the post-EFTR group (Table 2).

**Table 1:** **Results of endoscopic closure with the Overstitch™ suturing device**

	ESD	EFTR	Overall	*P*-value
Number of post-ESD and post-EFTR lesions, n	15	3	18	-
Primary closure success rate, % (n/N)	85.7 (12/14)	100.0 (3/3)	88.2 (15/17)	0.67
Mean maximum specimen diameter, mm	45.0	31.6	44.3	0.015
Median procedure time, min	15.5	74.0	20.0	0.017
Median number of stitches, n	2	4	3	0.005
Secondary closure success rate, % (n/N)	0 (0/12)	100 (3/3)	20 (3/15)	0.002

*Abbreviations*: ESD, endoscopic submucosal dissection; EFTR, endoscopic full-thickness resection.

**Table 2:** **Procedural complications**

	ESD	EFTR
Clinical symptoms, n		
Loss of appetite	0	0
Tarry stool	1	0
Drooling	0	0
Necropsy findings, n		
Surrounding organ injury	0	0
Abscess	0	2
Adhesion	0	2

*Abbreviations*: ESD, endoscopic submucosal dissection; EFTR, endoscopic full-thickness resection.

Procedural failure of primary closure only occurred in the first animal in the post-ESD group, even though post-EFTR defect closure was technically more challenging (especially regarding placement of the initial stitch to approximate the free ends of the full-thickness defect) than the mucosal apposition technique used for post-ESD ulcers. There were no significant clinical factors that accounted for the procedural difficulty in the two failed attempts. Despite practicing the procedure on *ex vivo* models prior to the *in vivo* study, there was still a learning curve for suture placement mainly because of the rigidity of the scope attached to the system.

On necropsy, minor abscesses and adhesions were observed in the post-EFTR group, but they were not associated with clinically significant symptoms during the 1-week survival period. Considering the inherent difficulty of preoperative preparation (i.e., achieving a sufficient fasting period) in pigs, we anticipate that the risk of severe peritoneal contamination could be lower in humans. However, the prophylactic use of antibiotics and careful clinical monitoring should be instituted during the perisurgical period.

To date, various types of endoscopic suturing devices have been developed, such as the EndoCinch (Davol Inc., Cranston, RI) [23], the Eagle Claw (Olympus Medical Systems Co.) [19], and the T-tag tissue apposition system (TAS; Ethicon Endo-Surgery Inc., Cincinnati, OH) [16,17,24–26]. Research on endoscopic suturing initially focused on the endoluminal creation of gastroplications as a minimally invasive treatment for gastroesophageal reflux disease and an alternative to surgical fundoplication. However, subsequent basic and clinical research studies have demonstrated that everted mucosal tissue apposition, which is the only form of achievable tissue apposition for endoluminal suturing in this situation, cannot be sustained long-term and that the plication eventually transforms into a flattened scar regardless of suturing depth and mucosal conditions (such as ischemia, ablation, and ulceration) [27,28].

ESD enables a large area of diseased mucosa to be radically removed from surrounding non-neoplastic tissues with repetitive needle knife dissection. The concept of en bloc resection by ESD is universally appreciated as desirable because it enables surgical excision of neoplastic lesions with “no touch isolation” to the extent that this is endoscopically possible; this has resulted in rapid adoption of the technique and its applications in all levels of the gastrointestinal tract. However, ESD has not been internationally accepted as a first-line therapeutic option because of the technical challenges associated with the procedure and the higher risk of adverse events such as bleeding, perforation, and delayed adverse events [29].

Various endoscopic suturing devices have been tested as novel tools in achieving reliable closure of post-ESD ulcers and avoiding these adverse events [15–17]. Kantsevoy and colleagues recently reported a case series of post-ESD ulcer closures using the OverStitch™ [15]. In their study, primary closure of post-ESD ulcers was achieved after removing specimens of over 30 mm in diameter (mean diameter, 42.5 ± 14.8 mm) in 12 out of 12 lesions (4 in the stomach and 8 in the colon). None of the patients experienced clinically significant adverse events. In our study, despite successful immediate closure (except during the inaugural training period), poor durability of mucosal apposition was observed in the post-ESD lesions, similar to results in previous gastroplication studies [27,28]. We anticipated that tissue ablation during repetitive needle knife dissection of the submucosa in ESD might trigger the healing process and help large ulcer floors to fold and fuse, although a previous anti-reflux study [27] denied any enhancement of tissue healing due to supplemental tissue ablation. However, all of the sutures superficially placed on the ulcer edges were brought up to the lumen by resulting foreign body reactions and the regenerative tissue elevation of the healing process, and all sutures were eventually dislodged without suture disruption.

There are possible explanations for the seeming discrepancy between the outcomes of this animal study and previously published initial clinical experience [15]. First, the sutures used in the clinical trial might have been dislodged, as in our study, but without detection; in the clinical trial, the first follow-up endoscopy was not performed until 3 months after the treatment, and residual sutures were only observed in 2 of 12 patients at that time. Temporary closure can provide effective coverage against adverse events for a couple of the most risky days; given the rarity of these events, the absence of any post-ESD adverse events in 12 lesions does not guarantee the durability of the sutures or the prophylactic effect of mucosal closure. Second, the porcine gastric wall and muscularis are much thicker than the human gut wall, and the tension on each suture caused by contractions of the porcine stomach may be much higher than that which occurs in the human stomach. The better durability of the sutures after EFTR observed in our study might be explained by the same theory. The loco-regional mechanical tension on the suture line may have been substantially reduced by disrupting the muscular fibers with EFTR, similar to the reduction in lower esophageal sphincter pressure seen after Heller’s myotomy in achalasia patients. Pathological analysis of ESD/EFTR lesions was only available in our porcine study; comparative pathological analysis might provide better evidence for the hypotheses above.

Given the inherent technical limitations of ER, EFTR is a long-awaited dream of all endoscopists: a technology that potentially eliminates the technical challenges currently associated with cleavage of the intramural layers and expands the indications for ER to include diseases arising from the deeper layers. The technical feasibility of post-EFTR closure using full-thickness endoscopic apposition devices has already been demonstrated, including in the clinical setting [8,17]. The tissue anchor-style suturing device is one of the endoscopic suturing systems that has been clinically tested [16,17,26] and shown to allow tissue approximation without limitation of the defect size [24]. This system has attracted much interest because of its procedural simplicity; full-thickness suture placement can be accomplished with simple, straight needle puncture under direct endoscopic observation [24]. However, the tissue anchoring device only creates mucosa-to-mucosa tissue apposition. Also, blind full-thickness needle puncture can give rise to inadvertent injury of surrounding organs. Therefore, in the limited clinical experience with full-thickness defect closure, the sutures have been placed with laparoscopic assistance [16].

The divergence between post-ESD and EFTR closure outcomes in this study clearly demonstrates that the form of tissue apposition (e.g., everted mucosa-to-mucosa, inverted serosa-to-serosa, or multi-layered) is more relevant to suture durability than other factors [30]. The size of the sampled specimens in the EFTR group was smaller than in the ESD group, and the stretchy ESD mucosal specimens could also be stretched larger than the full-thickness EFTR specimens when they were pinned out for size measurement. To eliminate the risks of post-operative extraluminal contamination during EFTR, a suturing device that permits inverted serosal apposition with minimal risk of injury to the surrounding organs should be used for defect closure. Although the development of an arbitrarily maneuverable, miniaturized, flexible surgical stapler enabling layer-to-layer suturing would be ideal, this remains technologically challenging [31]. Therefore, we believe that a device using a curved suturing needle, such as that used in this study, is currently the best available tool in achieving safe EFTR. However, beyond the closure technique, further technological improvement is still necessary to standardize the EFTR procedure and enable its broader application. Although we used T-bar-style tissue anchors to tent the anterior gastric wall and maintain intragastric working space during full-thickness tissue dissection and suturing, the application of this methodology is restricted and depends on the location of the lesion.

## Conclusions

This animal study demonstrated the feasibility and safety of the immediate closure of large post-ESD ulcers and post-EFTR defects with an endoscopic suturing device using a curved needle. However, mucosal apposition of post-ESD ulcers was not as durable as serosal apposition of post-EFTR defects. The outcome of this animal study clarifies the need for further investigation assessing the clinical benefits of closure versus “pouching” of large post-ER mucosal defects.

## Abbreviations

EFTR: Endoscopic full-thickness resection
ER: Endoscopic resection
ESD: Endoscopic submucosal dissection
